# Deep Learning With Anaphora Resolution for the Detection of Tweeters With Depression: Algorithm Development and Validation Study

**DOI:** 10.2196/19824

**Published:** 2021-08-06

**Authors:** Akkapon Wongkoblap, Miguel A Vadillo, Vasa Curcin

**Affiliations:** 1 Department of Informatics King's College London London United Kingdom; 2 DIGITECH Suranaree University of Technology Nakhon Ratchasima Thailand; 3 School of Information Technology Suranaree University of Technology Nakhon Ratchasima Thailand; 4 School of Population Health and Environmental Sciences King’s College London London United Kingdom; 5 Departamento de Psicología Básica Universidad Autónoma de Madrid Madrid Spain

**Keywords:** depression, mental health, Twitter, social media, deep learning, anaphora resolution, multiple-instance learning, depression markers

## Abstract

**Background:**

Mental health problems are widely recognized as a major public health challenge worldwide. This concern highlights the need to develop effective tools for detecting mental health disorders in the population. Social networks are a promising source of data wherein patients publish rich personal information that can be mined to extract valuable psychological cues; however, these data come with their own set of challenges, such as the need to disambiguate between statements about oneself and third parties. Traditionally, natural language processing techniques for social media have looked at text classifiers and user classification models separately, hence presenting a challenge for researchers who want to combine text sentiment and user sentiment analysis.

**Objective:**

The objective of this study is to develop a predictive model that can detect users with depression from Twitter posts and instantly identify textual content associated with mental health topics. The model can also address the problem of anaphoric resolution and highlight anaphoric interpretations.

**Methods:**

We retrieved the data set from Twitter by using a regular expression or stream of real-time tweets comprising 3682 users, of which 1983 self-declared their depression and 1699 declared no depression. Two multiple instance learning models were developed—one with and one without an anaphoric resolution encoder—to identify users with depression and highlight posts related to the mental health of the author. Several previously published models were applied to our data set, and their performance was compared with that of our models.

**Results:**

The maximum accuracy, F1 score, and area under the curve of our anaphoric resolution model were 92%, 92%, and 90%, respectively. The model outperformed alternative predictive models, which ranged from classical machine learning models to deep learning models.

**Conclusions:**

Our model with anaphoric resolution shows promising results when compared with other predictive models and provides valuable insights into textual content that is relevant to the mental health of the tweeter.

## Introduction

### Background

Mental health problems are widely recognized as major public health challenges worldwide. According to the World Health Organization, 264 million people were affected by depression globally in 2020 [1]. Mental illness, in general, is one of the leading causes of the global burden of this disease. It was estimated that in England, 105 billion British pounds (US $145 billion) were spent on mental health services and treatments or lost in productivity at work in 2018 [2], with the global costs expected to rise to US $6 trillion by 2030 [3]. A significant contributor to this cost is that people living with mental health problems sometimes receive inaccurate assessments [1]. This highlights the need for effective mental health services and a novel approach for diagnosing mental health disorders.

User-generated content on social media, reviews, blogs, and message board platforms offers an opportunity for researchers to explore and classify the huge amount of content in different domains, such as marketing [4], politics [5], and health [6-8], thereby providing a rapid method to understand user-created text and expressed emotion using text classification algorithms. Social networking (eg, Facebook and LinkedIn) and microblogging platforms (eg, Twitter and Tumblr) provide internet users with a safe space to post their feelings, thoughts, and activities. With some users publicly expressing their mental health statuses on their profiles, it becomes possible to train classification engines to detect internet users with mental health problems [9,10]. Using Twitter data, in particular, studies have examined users with depression [11-14], postpartum depression [15], anxiety, obsessive compulsive disorder, and posttraumatic stress disorder [11,16]. In addition, Facebook data were also used to detect users with depression [17,18] and postpartum depression [19].

Generally, text classifiers and user classification models tend to be developed separately. This presents a challenge for researchers who want to simultaneously understand both text sentiment analysis and user sentiment analysis. In this paper, we present a predictive model that can detect users with depression and identify their tweets as those related to health. An ideal technique for developing this type of model is multiple instance learning (MIL) [20], where the model can learn from a set of labeled bags or users instead of a set of individual instances or user-generated messages.

Anaphora resolution is an established natural language processing (NLP) problem and an emerging field in the analysis of social media content that helps with determining which previously mentioned person is the subject of a subsequent statement and understanding references to someone in the content on social media. This is particularly relevant to social media, as posts may frequently refer to individuals other than the tweeter [21].

### Objectives

To the best of our knowledge, no study has focused on detecting users with depression on social networks with an anaphoric interpretation of the content. In this study, we aim to address the problem of anaphora resolution in user-generated content and present a predictive model that can reliably identify statements, thoughts, and attitudes relating to the tweeter, rather than a third party.

The objective of this study is to investigate whether user-generated content from Twitter can be used to detect users with depression. This raises three research questions:

1. Can MIL be used to develop a predictive model for detecting users with depression from their tweets?
2. Can sentiments of unlabeled tweets be predicted from the labels of users with depression?
3. Can anaphora resolution be combined with MIL to eliminate false positives?

This paper introduces MIL models with and without anaphora resolution to detect users with depression from their generated textual content on Twitter and predictive models that can highlight posts relevant to mental health. The results show that our algorithm outperforms the major recently published algorithms in the field. We further illustrate the differences in the tweets related to mental health from users with self-declared depression and users with no depression.

### This Study

This study focuses on text analysis, predictive models for detecting social network users with mental disorders, and MIL. The most relevant studies published to date are reviewed below.

Text analysis is an NLP approach for identifying information within text. This technique has been developed to understand the textual content automatically and computationally. During the early stages of sentiment and emotion analysis, researchers manually annotated the text [22]. With the possibility of identifying emotions in text, the content has been computationally analyzed using a keyword or corpus-based approach and a learning-based approach [23,24].

The learning-based approach uses a predictive model to determine the relationship between an input and output word. Word embedding is a common learning-based technique that transforms the words of a document into dimensional vectors for word representation and determines word similarity. Global Vectors for Word Representation (GloVe) is a word-embedding approach that computes and aggregates word co-occurrence for representing the closest linguistic or semantic similarity between co-occurrent words as vectors [25]. GloVe was trained on several textual data sets, such as Wikipedia and common crawl (a copy of web content), and supported 50D, 100D, 200D, and 300D vectors.

Anaphora resolution is another text analysis problem related to determining which person is mentioned within textual content. There are three reference resolution algorithms [26]. The rule-based entity resolution extracts syntactic rules and semantic knowledge from the text. The statistical and machine learning–based entity resolution is a method to understand the coreference of a reference to an early entity. Deep learning for entity resolution reduces handcrafted feature requirements and represents words as vectors conveying semantic units. Aktaş et al [21] investigated anaphora resolution for conversations on Twitter using a corpus and manual annotation. Twitter conversations revealed the cues of anaphora resolution to identify a mentioned person and provide context.

De Choudhury and Gamon [13] pioneered NLP and machine learning approaches for developing predictive models to detect users with mental disorders from social network data using a mental health screening questionnaire and linguistic analysis tools to extract emotional words and web-based behaviors from users’ posts. However, the screening and data collection process was time consuming, and Coppersmith et al [11] introduced an automatic data gathering method using keywords to find the target users and programmatically retrieve the posts.

Following these initial studies, a number of novel methods have emerged for predicting mental disorders in social network users. The early work focused on classical supervised machine learning techniques and traditional text analysis approaches.

The psychometric analysis of textual content was used to compute the percentage of emotional, functional, and social concern words [13,15]. Linguistic inquiry and word count (LIWC) was used to compute the percentage of words relevant to categories from each tweet. The extracted percentages were then used to train a predictive model based on a support vector machine with a radial basis function [13].

Language models have been applied to analyze social media texts to address spelling errors, shortenings, and emoticons [11]. The language model was developed from an n-gram, which learns from the sequences of text and computes the probability of unseen text relevant to a category of the trained model. This model scored the probabilities of users with depression based on a higher probability of the positive class language model trained from the tweets of users with depression or the negative class language model developed from the tweets of control users [11].

A predictive model based on topic models was developed from the social network profiles of clinically diagnosed patients [17]. The topic model used latent Dirichlet allocation to extract topics from the text. All tweets from each user were used to compute 200 topics, which were then used to develop a logistic regression model for classifying the users with depression [17].

Building on the popularity of neural networks, novel models have been developed using word embedding [27,28] and deep neural network models [28]. The Usr2Vec model transformed text into an embedding matrix, where words commonly used together were represented in closely dimensional spaces for classifying users. The embeddings were learned from users’ tweets and then summarized as user representations. The embedding matrices were used to train a predictive model using a multinomial logistic regression technique [27].

The deep learning model uses word embeddings to represent the sequential words of users’ tweets. A predictive model was trained using a 1D convolutional neural network (CNN) and a global max pooling layer [28].

In addition to the textual content of the posts, a number of writing features can be analyzed: post or blog lengths, time gap between consecutive posts, and day of the week and time of the day of postings. Further network features of interest include likes, numbers of followers or following, characteristics of comments on other users’ posts compared with original posts, and numbers of shares or retweets. Image analysis was used to characterize user posts [29,30].

To develop a predictive model, this study focused on MIL. It is a supervised learning technique first proposed by Keeler et al [31,32]. Although classical supervised learning requires an instance 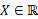 and a single label 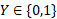 to learn during the training process, MIL can learn from a bag of instances *X=x_1_*, *x_2_*,…, *x_N_*. Each instance *x_n_* can be independent and has its own individual label, *y_n_,* where *y_n_* ∈ {0, 1} for *n*=1,…, *N*, and it is assumed that each *y_n_* is unknown during the training process. On the basis of these assumptions, an MIL classifier can predict a label *Y* for a given bag *X* as follows:



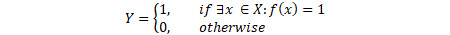



On the basis of these assumptions, MIL can provide an extreme result *Y*=1 in the case of having a predicted positive-instance label *y_n_*=1 in a given input *X.* The relaxation of the MIL assumption can be computed using aggregated probabilistic distributions of instances, where *Y=P(x_n_)* for *n*=1,…*, N*.

The purpose of MIL is to facilitate the development of a predictive model for detecting social media users with depression and instantly label each of the posts associated with either mental health or other topics. Normally, data sets from social networking are labeled at the user level but not at the post level. This makes it difficult to find a change in patterns in the message topics posted on social networks.

MIL models have been widely applied to image classification [32], object detection [33], image annotation [34], medical image and video analysis [35,36], sentence selection [37], and document classification [38]. In document sentiment analysis, Angelidis and Lapata [20] proposed the MIL network (MILNET) to classify web-based review documents and instantly identify the sentiment polarity of each segment of given documents. MILNET comprises segment encoding, segment classification, and document classification via an attention mechanism. Segment encoding transformed sentences in a document into segments via word-embedding matrices and a CNN. Each segment representation was classified using a softmax classifier. An attention mechanism based on a bidirectional gated recurrent unit (GRU) was used to weight the important segments to make a final document prediction as the weighted sum of the segment distributions. MILNET performed well in predicting the sentiment of a document and identifying the sentiment of the text segments but was not as successful in identifying a person mentioned in the document.

In this study, we adopt the MIL approach to develop two models, namely multiple instance learning for social network (MIL-SocNet) and multiple instance learning with an anaphoric resolution for social network (MILA-SocNet), to classify users with depression and highlight published posts associated with the mental health topic of a tweeter. Both models use novel document segment encoding, a tweet encoder, and user representation rather than a document vector. The latter model also includes the anaphora resolution, which further improves the performance.

## Methods

### Data Set

The data set was retrieved from Twitter, which provides an application programming interface (API) to search public tweets using regular expressions or stream real-time tweets. This study collected only tweets and users set as public. All collected tweets and users were anonymized. This study was approved by the King’s College Research Ethics Committee (reference number LRS-16/17-4705).

We selected a group of users with depression using the method proposed by Coppersmith et al [11]. Specifically, a regular expression was used to search tweets that contained the statement “I was diagnosed with depression” between January and May 2019. This resulted in 4892 tweets from 4545 unique users, who were then manually screened to ensure that the tweets did not refer to jokes, quotes, or someone else’s depression symptoms. After removing these messages, all tweets in the profiles of the users who posted the tweets were downloaded. After verification, 2132 unique users were included in this data set.

A control group was randomly selected from a list of 2036 users who posted tweets between June 1 and June 7, 2019. Users from the group with depression were removed from the list of the control group.

The limits imposed by the Twitter API allowed us to only download the 3200 most recent tweets of all verified users from the depressed and control groups. In total, 5 million tweets were collected from the 2132 users with depression and 4.2 million tweets from the 2036 users with no declared depression.

### Preprocessing

**Figure 1:**
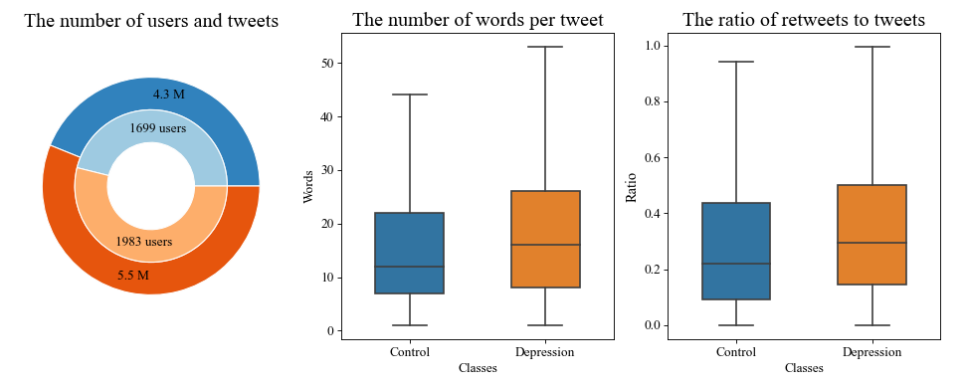
Analysis of data set statistics. The left side shows the percentages of users and tweets between control users and users with depression, where the inner circle presents the number of users and the outer circle presents the number of posts. The middle shows the number of words per post between 2 groups. The right side shows the ratio of retweets to tweets per user between the classes. Blue denotes the control group, and orange represents the depressed group.

**Figure 2:**
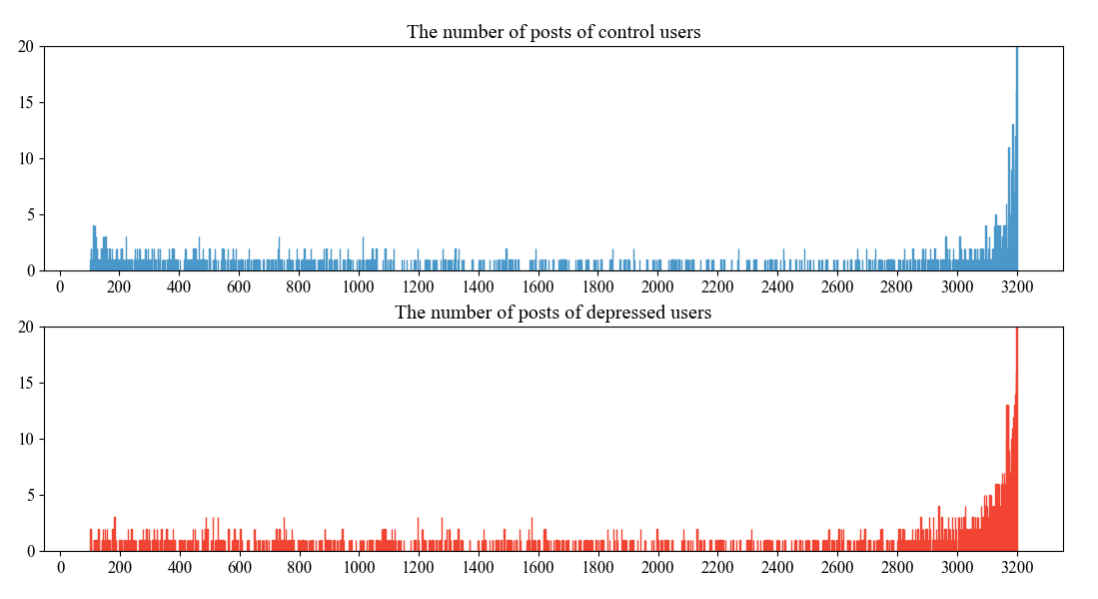
The distribution of the number of tweets between the depressed and control groups. This only shows a maximum of 20 tweets for clarity. The depression group with 3200 tweets had 436 users, and the control group with 3200 tweets had 485 users.

Before developing our MIL model, several transformations were performed on the data set. First, the user ID in each tweet was replaced by a generic *user*. Similarly, any numbers mentioned in tweets were replaced by the *number* and any specific URLs by *url*. The # character in each hashtag was replaced by the string *hashtag* (eg, *#depression* became *hashtag depression*). Finally, users with fewer than 100 tweets or less than 80% of tweets in English were removed from the data set, resulting in 3682 users, 1983 with declared depression and 1699 with no declared depression, as depicted in on the left-hand side of Figure 1. In addition, other dimensions of the data set were explored, as shown in Figure 1. Figure 2 illustrates the distribution of the number of tweets between the depressed and control groups. Slight differences were present between the control and depressed groups.

All tweets in our final data set were embedded from pretrained GloVe word vectors. GloVe is an unsupervised machine learning approach and an NLP technique that represents a word as a set of word vectors. GloVe computes and aggregates word co-occurrences to create a vector representation of the closest linguistic or semantic similarity between co-occurrent words [22]. As explained earlier, GloVe was trained on several textual data sets, for example, Wikipedia and common crawl (a copy of web content), and supported 50D, 100D, 200D, and 300D vectors. However, our study used pretrained word vectors trained on 2 billion tweets and 100D vectors to transform our tweets into word embedding.

### Predictive Model

#### Overview

This section describes the structure of our predictive model to classify a Twitter user with depression. This section will explain how an MIL model with supervised neural networks classifies users and provides a changing pattern of generated text associated with mental health or other topics.

**Figure 3:**
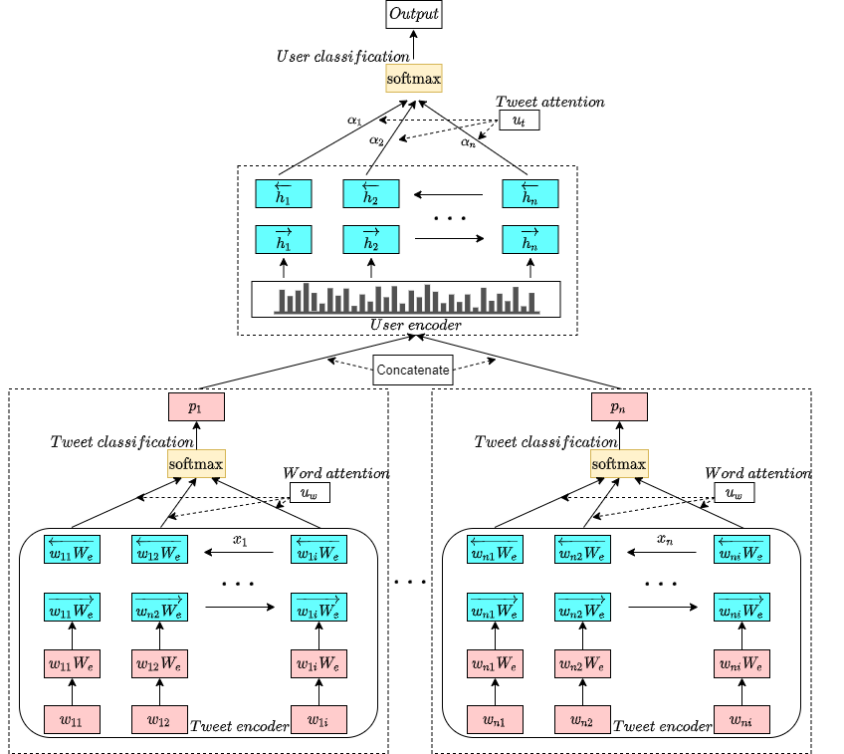
The structure of our proposed multiple instance learning-SocNet.

Our proposed MIL-SocNet architecture comprises a tweet encoder, word attention on a tweet, tweet classification, a user encoder, tweet attention, and user classification (Figure 3). The differences between MIL-SocNet and the basic MILNET architecture are the tweet encoder and word attention, respectively. Our model uses a GRU, whereas MILNET uses a CNN and does not have an attentional mechanism.

**Figure 4:**
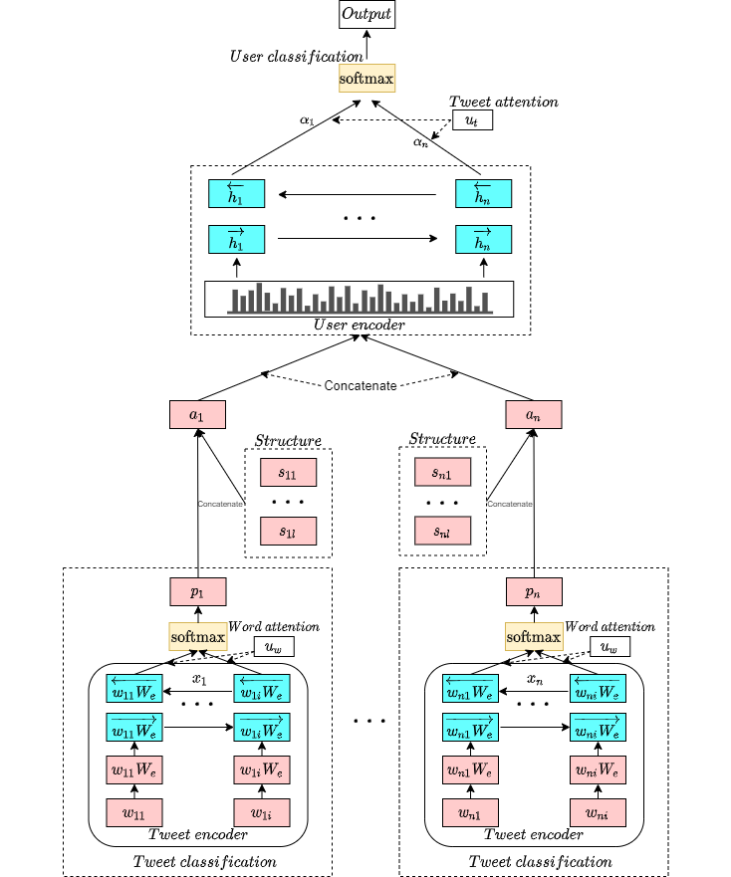
The structure of our proposed MILA-SocNet.

Furthermore, the MIL-SocNet model was extended with an anaphoric resolution to create the MILA-SocNet model. We present this model to improve performance by adding an anaphora resolution encoder to ensure that the algorithm focuses on posts related to the author (Figure 4).

#### Tweet Encoder

The first layer of our proposed model transforms each tweet into a machine-readable form. First, tweets were transformed into word-embedding matrices. Each user publishes *j*=1, 2,…, *n* tweets, where *n* is the number of tweets used to train the model. Each tweet contains *k*=1, 2,…, *i* words, where *i* is the number of words in each tweet and varies from post to post. *W_jk_* represents the *k*th word in the *j*th tweet. Every *w_jk_* is then embedded through an embedding matrix *W_e_* to be received a word vector *x_jk_*. This layer embeds all words *w_jk_* of jth post to the word vector:

*x_jk_* = *w_jk_W _e_*, *j* ∈ [1, *n*] and *k* ∈ [1, *i*]

The abovementioned equation operates 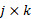 times. After embedding all words, a bidirectional GRU is used to encode the vector:



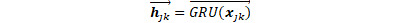





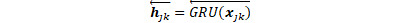





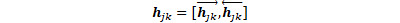



The bidirectional GRU presents a hidden representation of *h_jk_*, which is concatenated from 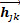 and 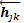. The word hidden vector h_jk_ is then sent to an attention mechanism to select the important words.

#### Word Attention on a Tweet

Not every word equally represents tweet meanings. An attention mechanism is used to select words that best capture the relevant meaning of a tweet. The attention layer comprises a tanh function to produce an attention vector *u_jk_* of the *k*th word in the *j*th tweet, where *W_w_* and *b_w_* are weights and bias, respectively.

*u_jk_* = *tanh* (*W_w_h_jk_* + *b_w_*)

The importance of words or attention weights *a_jk_* is calculated via the normalized similarity of *u_jk_* with the context vector of the word level *u_w_*, which is learned and updated during the training step.



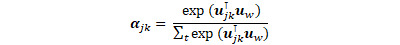



Finally, the tweet vector *t_j_* is computed using the weighted sum of word importance with the hidden representation of *h_jk_* generated from the bidirectional GRU.



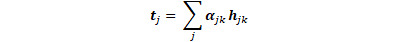



#### Tweet Classification

To make a prediction about a tweet related to either a mental health or another topic, each tweet vector *t*_1_, *t*_2_,..., *t*_n_ from the attention layer is classified through a softmax function [39].



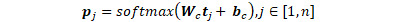



The function generates the probabilities of tweet labels 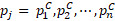, where 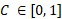 with 1 denoting a mental health–related post and 0 denoting a non–mental health–related post. The labels used to train this layer are derived and computed from the labels of the user level only. The parameters *W*_c_ and *b*_c_ are learned and updated during the training step. Every predicted tweet label is used to teach a predictive model and detect a user with depression.

#### User Encoder

Detecting users with depression requires a pattern to differentiate between user groups. To predict these users, this study used a temporal pattern of posting generated from the tweet classification layer. This layer concatenates the probabilities of every classified tweet label into a single list of label probabilities called *user representation*. The user representations between the 2 groups are expected to differ, which will be explored and illustrated in the Discussion section. Then, user representation is passed through a bidirectional GRU to learn the changing patterns of text categories over the observation time. This generates the forward hidden state 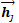 and the backward hidden state 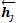 of the user representation. Finally, they were concatenated to *h_j_*.



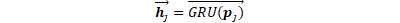





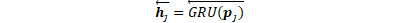





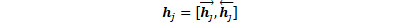



#### Anaphora Resolution Encoder

For the MILA-SocNet model with anaphora resolution, pronoun features from LIWC [40] are used to add informative interpretations to each tweet. Every tweet was analyzed for emotions, thinking styles, social states, parts of speech, and psychological dimensions.

Each tweet is combined between the extracted pronoun features *s_j_* from the LIWC and a tweet classified label *p_j_* from the tweet classification layer, where 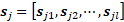 with 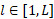 represents the extracted features in the *j*th tweet. This yields the following anaphora resolution vector:



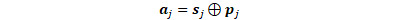



The vector is then passed through a bidirectional GRU to learn the text category and anaphoric features. This generates *h_j_* combined from the forward hidden state 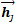 and backward hidden state 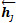.



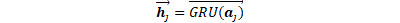





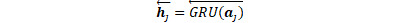





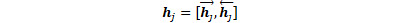



#### Tweet Attention

Not all user tweets were equally associated with depression. Some tweets may contain cues relevant to depression, whereas others may not. For this purpose, an attention mechanism is applied to reward tweets that correctly represent the characteristics and are important for correctly detecting a user with depression. This layer performs similarly in both MIL-SocNet and MILA-SocNet. A multilayer perceptron (MLP) produces the attention vector *u_j_* of the *j*th tweet. The parameter *W_t_* denotes the weights of the tweet and parameter *b_t_* represents the bias of the tweet.



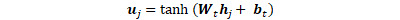



The attention weights of tweets or important tweets *α_j_* are computed through the similarity of *u_j_* with the context vector of tweet level *u_t_*, which is learned and updated during the training step.



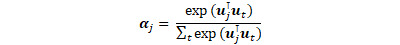



The user vector *v* is achieved by summarizing all the information of the tweet label possibilities of a user.



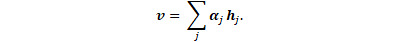



#### User Classification

Finally, a predictive model for detecting a user with depression can be achieved through the user vector *v* derived from encoding the concatenation of the probabilities and the attention weights of the classified tweet labels from the user. A softmax function was again used to perform the classification.



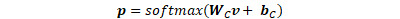



### Training the MIL Model

To train MILA-SocNet and MIL-SocNet, we used the Keras library with TensorFlow backend, a Python library for neural network APIs. We used an adaptive and momental bound method (AdaMod) [41], and the binary cross-entropy loss function to minimize loss. Every tweet from each user was tokenized and limited to 55 tokens or words. The model was trained using 2000 recent tweets from each user, with users with fewer than 2000 tweets having empty tweets padded with 0 values to achieve the matching length. To eliminate overfitting, dropout and early stopping were applied to the model during the training step.

**Figure 5:**
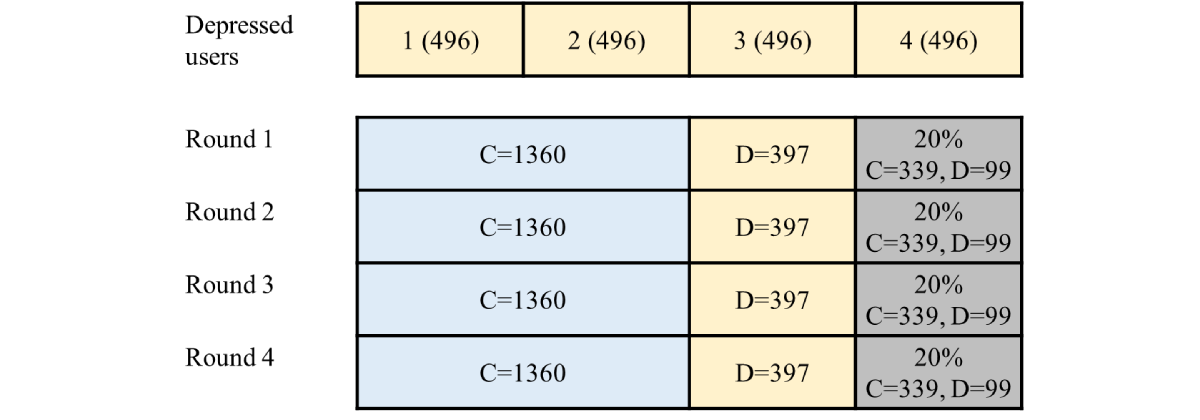
Holdout cross-validation on our experiment. C denotes control users and D represents users with depression. Blue, yellow, and gray represent control data, chunks of users with depression, and test sets, respectively.

Both our models and replicated models were trained and tested with holdout cross-validation. We split the users experiencing depression into four equal chunks and trained the models against all control users. Thus, each round used 496 users experiencing depression (22.60%) and 1699 control users (77.40%), mirroring the real-world incidence of depression. From the total users included in each round, 20% were used as test sets to evaluate the performance of the models. To reserve the same proportions of classes between the training and test tests, stratified cross-validation was used. Figure 5 shows the cross-validation process.

### Model Evaluation

To predict whether each Twitter user was likely to be depressed, we also trained a set of published predictive models ranging from classical machine learning to deep learning techniques by using user-generated textual content. Accuracy, precision, recall, and F1 scores were averaged across the test sets. Each model was trained and tested with the same samples in each round; however, data transformations differed in some cases, as explained in the Background section.

To compute the predictive performance of models for detecting social network users with depression, we used the following metrics:



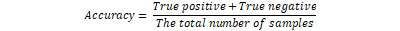





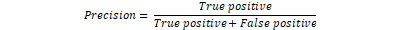





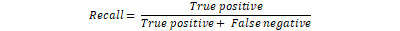





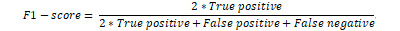



To further compare the performance of MILA-SocNet and MIL-SocNet with the other published models, Akaike information criterion (AIC) was applied across all the models. AIC is a commonly used tool for model comparison and selection [42,43] that measures the information loss in each model, considering the model’s complexity as well. AIC is defined as follows:



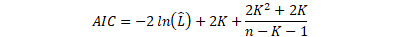



where *n* is the number of samples and *K* is the number of parameters or features of a model. 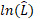 denotes the natural logarithm of likelihood [44]. The equation also uses bias adjustment because of the small sample size [45,46]. A lower AIC value indicates better performance.

## Results

This section shows the performance of MILA-SocNet and MIL-SocNet and compares their results in terms of accuracy, precision, recall, and F1 score against several published models including LIWC [13], language [11], topic [17], Usr2Vec [27], and deep learning [28] models, as explained in the Background section.

**Figure 6:**
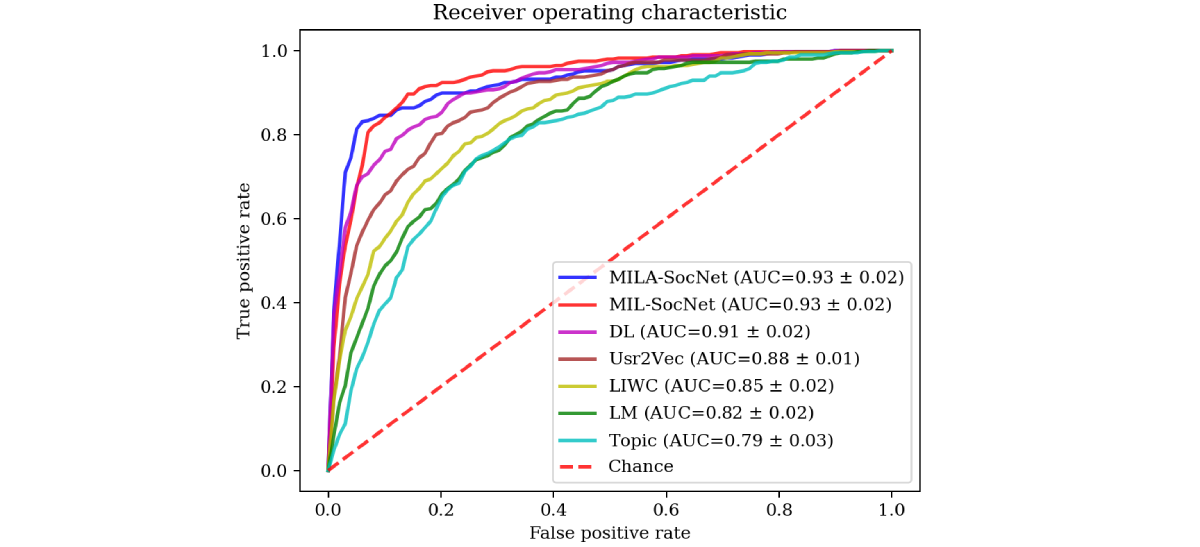
Receiver operating characteristic curves of each model. Area under the curve with SDs of each model are denoted by different colors. The x-axis shows the false-positive rate, and the y-axis presents the true-positive rate. The dashed line indicates a random guess. AUC: area under receiver operating curve; DL: deep learning model; LIWC: linguistic inquiry and word count; LM: language model.

Table 1 shows the performance of our proposed MILA-SocNet and MIL-SocNet models against the alternative models. As observed, the MILA-SocNet achieves a maximum accuracy (92%), precision (92%), recall (92%), and F1 score (92%), immediately followed by the MIL-SocNet. The MIL-SocNet yielded an accuracy, precision, recall, and F1 score of 90%, 91%, 90%, and 90%, respectively. Each model was evaluated using the area under the curve of the receiver operating characteristic curve. As can be seen in Figure 6, the MILA-SocNet and MIL-SocNet models achieved the highest areas under the curve—93% in both cases. It should be noted that in those studies, the replicated models were reported with different proportions of classes. These results might be higher or lower than our reported results. In our study, the baseline result was 77% in the case of predicting the majority class in all cases. As can be observed, all the models achieved results that were above this baseline.

Table 2 lists the AIC values for each model. The likelihood was computed from the model-based probabilities of the observed labels. The number of parameters of the MILA-SocNet, MIL-SocNet, and deep learning models were recovered from the number of trainable parameters reported by the Keras library. The number of parameters of the language model was taken from the number of vocabularies in the positive and negative language models. The number of parameters of LIWC, Usr2Vec, and topic models were features in the models. The likelihoods and AICs were averaged from cross-validation, as explained earlier. As can be observed, MILA-SocNet achieves the lowest AIC, reflecting the best performance.

**Table 1:** Performance of our proposed MILA-SocNet (multiple instance learning with an anaphoric resolution for social network) and MIL-SocNet (multiple instance learning for social network) models and all replicated models.

Model	Accuracy, %	Precision	Recall	F1 score
MILA-SocNet	92.14	0.92	0.92	0.92
MIL-SocNet	90.49	0.91	0.90	0.90
Deep learning	89.07	0.89	0.89	0.89
Usr2Vec	84.38	0.84	0.84	0.83
LIWC^a^	83.31	0.83	0.83	0.81
Language	81.61	0.80	0.82	0.79
Topic	80.13	0.78	0.80	0.78

^a^LIWC: linguistic inquiry and word count.

**Table 2:** The Akaike information criterion (AIC) results against all models. Each row is reported with the number of parameters (K), the residual sum of squares, and the AIC. A lower AIC is better.

Model	Number of parameters, K	Likelihood	AIC
MILA-SocNet^a^	59,668	−143.72	−597.05
MIL-SocNet^b^	56,296	−210.22	−464.45
Deep learning	138,502	−309.97	−260.84
Language	16695.5	−420.31	−61.03
LIWC^c^	93	−169.62	575.92
Usr2Vec	100	−190.28	640.32
Topic	200	−276.42	1290.66

^a^MILA-SocNet: multiple instance learning with an anaphoric resolution for social network.

^b^MIL-SocNet: multiple instance learning for social network.

^c^LIWC: linguistic inquiry and word count.

## Discussion

### Principal Findings

In this study, we presented two novel MIL models for detecting social network users with depression based on their self-identifying tweets. The original MIL-SocNet model was extended with anaphoric resolution to produce the second MILA-SocNet model. We also compared the performance of both models with that of several previously published models. As can be seen from Tables 1 and 2, MILA-SocNet and MIL-SocNet outperformed all other models in all metrics. We now look at several potential reasons for this result.

Although deep learning models can be trained on raw textual data, traditional machine learning models (eg, the LIWC, language, topic, and Usr2Vec models) require feature extraction to be performed using external tools, which may introduce the additional risk of losing useful information from short textual data [47,48]. For instance, misspelled and abbreviated words in tweets may not be present in the dictionary of an extraction tool, resulting in the mislabeling of words. This may be one of the reasons why traditional machine learning techniques performed worse than our proposed models.

Another reason for the performance gap may be that the sequential ordering of words in a tweet and tweets posted on a timeline may influence model performance. Training a predictive model with traditional machine learning methods requires aggregated data, which may cause the loss of contextual information compared with deep neural networks that can learn from the sequential information in the data [49-52].

Unlike the deep learning model that we have compared against [28], MILA-SocNet and MIL-SocNet used an attention mechanism that highlights words and tweets relevant to mental health. This attention mechanism may have contributed to our proposed models outperforming the deep learning model, even though our approach is also based on deep learning techniques.

Another important point to consider is that the addition of anaphoric resolution improves the performance of the base MIL model. The difference between MILA-SocNet and MIL-SocNet is only in anaphora resolution encoding, which highlights posts related to the tweeters rather than someone else. This is an important feature that has not been widely investigated in the field and should be considered while designing future studies.

**Figure 7:**
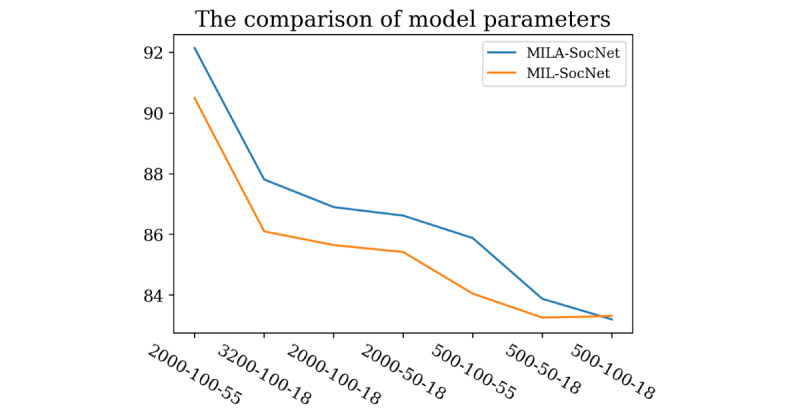
Results from different model parameters. Y-axis is the accuracy of the models. X-axis represents the number of posts, embedded dimensions, and post tokens in each model.

We further explored our proposed models by comparing the model performance under different conditions. A set of different parameters was used to train the models. The number of each user’s posts used to train a model ranged from 500 to 3200 posts. The numbers of embedded dimensions were 50 and 100. The lengths of word tokens used to train the models were 18 and 55 tokens, respectively. Table 3 and Figure 7 show the predictive results of MILA-SocNet and MIL-SocNet with different parameters. Longer post length and longer word token provide better results, which is expected as these provide more textual content. Furthermore, models with fewer embedded dimensions perform worse than models with more dimensions.

After training the models, we investigated their interpretability by observing the attention weights to find out which tweets the model paid most attention to. Two users from each group were randomly selected from those correctly labeled by our model, and attention weights were extracted from the tweet attention layer. Textbox 1 highlights the tweets that achieved the highest and lowest weights for these 4 users, offering some insight into model’s decision-making. Our predictive model with anaphoric resolution can identify tweets related to the tweeters’ own experiences.

**Table 3:** Performance of MILA-SocNet (multiple instance learning with an anaphoric resolution for social network) and MIL-SocNet (multiple instance learning for social network) with different parameters. The first number in the model name (first column) represents the number of posts, the second is the number of embedded dimensions, and the last is the number of word tokens.

Model name	MILA-SocNet models				MIL-SocNet models			
	Accuracy, %	Precision	Recall	F1 score	Accuracy, %	Precision	Recall	F1 score
2000-100-55	92.14	0.92	0.92	0.92	90.49	0.91	0.90	0.90
500-100-55	85.88	0.86	0.86	0.84	84.05	0.83	0.84	0.83
3200-100-18	87.81	0.87	0.88	0.88	86.10	0.85	0.86	0.86
2000-100-18	86.90	0.86	0.87	0.86	85.65	0.85	0.86	0.85
500-100-18	83.20	0.82	0.83	0.82	83.31	0.83	0.83	0.81
2000-50-18	86.62	0.86	0.87	0.86	85.42	0.85	0.85	0.85
500-50-18	83.88	0.83	0.84	0.83	83.26	0.83	0.83	0.82

**Attention weights of posts. The “text” was paraphrased to anonymize users’ identities.**

**Users with depression**

- User 1
  Highest weight: I was also dealing with depression and anxiety badly. School was hell.
  Lowest weight: @user Exam without someone’s supervision is bad.
- User 2
  Highest weight: I get some rest, take medication, and engage with what I like. These help me and I do not force myself to do things.
  Lowest weight: Talk about offensive things to physical harm: url.

**Users with no depression**

- User 1
  Highest weight: The lady christmas jumper: url.
  Lowest weight: All the best for your match and hope to see you play.
- User 2
  Highest weight: He reminds me someone in a football team. He can play many positions and he is our best player.
  Lowest weight: People believe you when you have evidence.

A recent survey on using social media data to identify users with depression showed that users from the United Kingdom expressed serious concerns about privacy risks and did not see the potential societal benefits outweighing these risks [53]. Thus, if these technologies are to have a meaningful impact on people’s lives, increased importance must be placed on the transparency and trust of the analytics performed.

Achieving this trust is, to an extent, helped by the compliance of any research with ethical codes and with the General Data Protection Regulation (GDPR), which helps in raising confidence in data safety and transparent analysis. However, *GDPR Article 9: Processing of special categories of personal data* specifically mentions that consent is not required if permission relates to personal data that are manifestly made public by the data subject. A core problem is the perception that any data in the public domain are automatically available for research. This is highly controversial from an ethical point of view, as the disruption presented by the wide availability of social network data impacts the norms that guide our perception of the usage of our data for research. Ultimately, GDPR is focused on process, not on the *objective* of the research, which is fundamental to shaping any research consent and the social consensus around it.

This study had some limitations. Collecting control group data is challenging because the samples may contain users with depression who do not publicly express their mental health state on their profiles. Although keyword-based self-declaration is a popular way of asserting depression [11,12], social media users with depression may use more complex ways of communicating their mental health state [54]. There is evidence that social media users post less frequently when they feel low, suggesting that there may be less data available for modeling depression [53].

With regard to technical limitations, this study used additional features from a language analysis tool, which counts words in psychological and word function categories. This may prevent our models from learning word functions directly from sentences. Our future work will use sentence structures extracted from text and train a predictive model with those features [55], which may produce further performance improvements.

The availability of data for model validation is another major concern. Owing to potential ethical issues, there are currently no open data sets to evaluate the performance of predictive models on social network data, making it difficult to compare the model performance. The alternative benchmarking approach used in this study is to replicate well-known study models in the field and apply them to the same data set as the new model being investigated.

Another source of potential bias is the pages that publish tweets about mental health information (eg, mental health charities) and users who report depression experiences of other people (eg, users’ friends, family, or a celebrity). Although we filtered those instances in our study, a significant concern still exists for similar work in the field.

### Conclusions

This paper proposes two novel MIL models with and without anaphoric resolution to detect Twitter users with depression. Anaphoric resolution is introduced to address the problem of identifying the subject of a statement made in the post. The classifiers developed comprise a tweet encoder, word attention, tweet classification, user encoder, anaphoric resolution encoder, tweet attention, and user classification layers. Bidirectional long short-term memory layers were used to learn the sequence of words and order of tweets posted on a timeline. Word embedding was applied to transform the textual content into vectors. Additional pronoun features were used to add informative dimensions to our proposed model and highlight posts relevant to the posters themselves. The approach was evaluated against previously published traditional machine learning and deep learning techniques, and the experimental results show that our proposed model produces notably better results. Anaphoric resolution, in particular, improved the performance of our model further and should be considered for inclusion in future studies.

The potential impact of this research lies in its ability to offer social media users exhibiting signs of depression that are suitable for their formal diagnosis. As in other mental health disorders, treatments for depression produce better outcomes and at a lower cost of treatment, the earlier patients get into services. Targeted advertising by mental health charities may be seen as intrusive but is no different than companies advertising any other products to potential consumers based on their web activity.

Early research into public perception of this type of data usage shows that there is public skepticism about this approach. To overcome this animosity toward using social media data for mental health prediction modeling, we believe that future research in this area should focus on explainability and interpretability. We have shown that deep learning MIL models perform well, but they offer no explanation of their decision-making processes [56,57]. Extraction of patterns from the models can provide interpretability, as we demonstrated with tweet weight examples, and systematic sampling should be used to achieve the levels of trust acceptable to users. To gauge how acceptable these techniques are to the public, we intend to work with citizen juries to explore the change in opinion that such explainability can deliver [58].

## Abbreviations

AIC: Akaike information criterion
API: application programming interface
CNN: convolutional neural network
GDPR: General Data Protection Regulation
GloVe: Global Vectors for Word Representation
GRU: gated recurrent unit
LIWC: linguistic inquiry and word count
MIL: multiple instance learning
MILA-SocNet: multiple instance learning with an anaphoric resolution for social network
MILNET: multiple instance learning network
MIL-SocNet: multiple instance learning for social network
NLP: natural language processing

## References

[1] Depression. World Health Organization.

[2] Health Matters: Reducing Health Inequalities in Mental Illness. Public Health England.

[3] (2011). The Global Economic Burden of Noncommunicable Diseases. World Economic Forum.

[4] Lee JH, Jung SH, Park J (2017). The role of entropy of review text sentiments on online WOM and movie box office sales. Electr Comm Res Appl.

[5] Wilkerson J, Casas A (2017). Large-scale computerized text analysis in political science: opportunities and challenges. Annu Rev Polit Sci.

[6] Korkontzelos I, Nikfarjam A, Shardlow M, Sarker A, Ananiadou S, Gonzalez GH (2016). Analysis of the effect of sentiment analysis on extracting adverse drug reactions from tweets and forum posts. J Biomed Inform.

[7] Ive J, Gkotsis G, Dutta R, Stewart R, Velupillai S (2018). Hierarchical Neural Model With Attention Mechanisms for the Classification of Social Media Text Related to Mental Health. Proceedings of the Fifth Workshop on Computational Linguistics and Clinical Psychology: From Keyboard to Clinic.

[8] Gkotsis G, Oellrich A, Velupillai S, Liakata M, Hubbard TJ, Dobson RJ, Dutta R (2017). Characterisation of mental health conditions in social media using Informed Deep Learning. Sci Rep.

[9] Wongkoblap A, Vadillo MA, Curcin V (2017). Researching mental health disorders in the era of social media: systematic review. J Med Internet Res.

[10] Reece AG, Danforth CM (2017). Instagram photos reveal predictive markers of depression. EPJ Data Sci.

[11] Coppersmith G, Dredze M, Harman C (2014). Quantifying Mental Health Signals in Twitter. the Workshop on Computational Linguistics and Clinical Psychology: From Linguistic Signal to Clinical Reality.

[12] Yazdavar A H, Al-Olimat H S, Ebrahimi M, Bajaj G, Banerjee T, Thirunarayan K, Pathak J, Sheth A (2017). Semi-Supervised Approach to Monitoring Clinical Depressive Symptoms in Social Media. http://europepmc.org/abstract/MED/29707701.

[13] De Choudhury M, Gamon M, Counts S, Horvitz E (2013). Predicting Depression via Social Media. the International AAAI Conference on Web and Social Media.

[14] Leis A, Ronzano F, Mayer MA, Furlong LI, Sanz F (2019). Detecting Signs of Depression in Tweets in Spanish: Behavioral and Linguistic Analysis. J Med Internet Res.

[15] De Choudhury M, Counts S, Horvitz E (2013). Predicting postpartum changes in emotion and behavior via social media.

[16] Coppersmith G, Dredze M, Harman C, Hollingshead K (2015). From ADHD to SAD: Analyzing the Language of Mental Health on Twitter through Self-Reported Diagnoses.

[17] Eichstaedt JC, Smith RJ, Merchant RM, Ungar LH, Crutchley P, Preoţiuc-Pietro D, Asch DA, Schwartz HA (2018). Facebook language predicts depression in medical records. Proc Natl Acad Sci U S A.

[18] Schwartz H. A., Eichstaedt J, Kern M. L., Park G, Sap M, Stillwell D, Kosinski M, Ungar L (2014). Towards Assessing Changes in Degree of Depression through Facebook.

[19] De Choudhury M, Counts S., Horvitz E., Hoff A. (2014). Characterizing Predicting Postpartum Depression from Shared Facebook Data.

[20] Angelidis S, Lapata M (2018). Multiple Instance Learning Networks for Fine-Grained Sentiment Analysis. TACL.

[21] Aktaş B., Scheffler T., Stede M. (2018). Anaphora Resolution for Twitter Conversations: An Exploratory Study.

[22] Mohammad S. M., Meiselman H. L. (2016). 9 - Sentiment Analysis: Detecting Valence, Emotions, and Other Affectual States from Text. Emotion Measurement.

[23] Kim E, Klinger R (2018). A Survey on Sentiment and Emotion Analysis for Computational Literary Studies.

[24] Al-Saqqa S, Abdel-Nabi H, Awajan A (2018). A Survey of Textual Emotion Detection.

[25] Pennington J, Socher R, Manning C (2014). GloVe: Global Vectors for Word Representation.

[26] Sukthanker R, Poria S, Cambria E, Thirunavukarasu R (2020). Anaphora and coreference resolution: a review. Inform Fusion.

[27] Amir S, Coppersmith G, Carvalho P, Silva M J, Wallace B C (2017). Quantifying Mental Health from Social Media with Neural User Embeddings.

[28] Orabi A H, Buddhitha P, Orabi M H, Inkpen D (2018). Deep Learning for Depression Detection of Twitter Users.

[29] Kang K, Yoon C, Kim E Y (2016). Identifying depressive users in Twitter using multimodal analysis.

[30] Chancellor S, Lin Z, Goodman E L, Zerwas S, De Choudhury M (2016). Quantifying and Predicting Mental Illness Severity in Online Pro-Eating Disorder Communities.

[31] Keeler J, Rumelhart D, Leow W (1991). Integrated Segmentation and Recognition of Hand-Printed Numerals. Advances in Neural Information Processing Systems.

[32] Ilse M, Tomczak J M, Welling M (2018). Attention-based Deep Multiple Instance Learning.

[33] Cinbis RG, Verbeek J, Schmid C (2017). Weakly Supervised Object Localization with Multi-Fold Multiple Instance Learning. IEEE Trans. Pattern Anal. Mach. Intell.

[34] Wu J, Yu Y, Huang C, Yu K (2015). Deep multiple instance learning for image classification and auto-annotation.

[35] Xu Y, Mo T, Feng Q, Zhong P, Lai M, Chang E (2014). Deep learning of feature representation with multiple instance learning for medical image analysis. Yan Xu; Tao Mo; Qiwei Feng; Peilin Zhong; Maode Lai; Eric I-Chao Chang.

[36] Quellec G, Cazuguel G, Cochener B, Lamard M (2017). Multiple-Instance Learning for Medical Image and Video Analysis. IEEE Rev. Biomed. Eng.

[37] Wang W, Ning Y, Rangwala H, Ramakrishnan N (2016). A Multiple Instance Learning Framework for Identifying Key Sentences and Detecting Events.

[38] Yan S, Zhu X, Liu G, Wu J (2016). Sparse multiple instance learning as document classification. Multimed Tools Appl.

[39] Bishop C (2006). Pattern Recognition and Machine Learning.

[40] Tausczik YR, Pennebaker JW (2009). The Psychological Meaning of Words: LIWC and Computerized Text Analysis Methods. Journal of Language and Social Psychology.

[41] Kingma D P, Ba J (2014). Adam: A Method for Stochastic Optimization.

[42] Hauenstein S, Wood SN, Dormann CF (2017). Computing AIC for black-box models using generalized degrees of freedom: A comparison with cross-validation. Communications in Statistics - Simulation and Computation.

[43] Vrieze SI (2012). Model selection and psychological theory: A discussion of the differences between the Akaike information criterion (AIC) and the Bayesian information criterion (BIC). Psychological Methods.

[44] Akaike H (1974). A new look at the statistical model identification. IEEE Trans. Automat. Contr.

[45] Panchal G, Ganatra A, Kosta Y, Panchal D (2010). Searching Most Efficient Neural Network Architecture Using Akaike's Information Criterion (AIC). IJCA.

[46] HURVICH CM, TSAI C (1989). Regression and time series model selection in small samples. Biometrika.

[47] Wang J, Wang Z, Zhang D, Yan J (2017). Combining Knowledge with Deep Convolutional Neural Networks for Short Text Classification.

[48] Uysal A, Murphey Y (2017). Sentiment Classification: Feature Selection Based Approaches Versus Deep Learning.

[49] Lee J, Dernoncourt F (2016). Sequential Short-Text Classification with Recurrent and Convolutional Neural Networks.

[50] Lai S, Xu L, Liu K, Zhao J (2015). Recurrent convolutional neural networks for text classification.

[51] Zhang X, Zhao J, LeCun Y (2015). Character-level convolutional networks for text classification.

[52] Wang P, Xu B, Xu J, Tian G, Liu C, Hao H (2016). Semantic expansion using word embedding clustering and convolutional neural network for improving short text classification. Neurocomputing.

[53] Ford E, Curlewis K, Wongkoblap A, Curcin V (2019). Public Opinions on Using Social Media Content to Identify Users With Depression and Target Mental Health Care Advertising: Mixed Methods Survey. JMIR Ment Health.

[54] Berry N, Lobban F, Belousov M, Emsley R, Nenadic G, Bucci S (2017). #WhyWeTweetMH: Understanding Why People Use Twitter to Discuss Mental Health Problems. J Med Internet Res.

[55] Li J, Luong T, Jurafsky D, Hovy E (2015). When Are Tree Structures Necessary for Deep Learning of Representations?.

[56] Holzinger A, Biemann C, Pattichis C S, Kell D B (2017). What do we need to build explainable AI systems for the medical domain?. arXiv.

[57] Gilpin LH, Bau D, Yuan Z, Bajwa A, Specter M, Kagal L (2018). Explaining Explanations: An Overview of Interpretability of Machine Learning.

[58] Tully MP, Hassan L, Oswald M, Ainsworth J (2019). Commercial use of health data-A public “trial” by citizens' jury. Learn Health Sys.

